# Non-linear Amplification of Variability Through Interaction Across Scales Supports Greater Accuracy in Manual Aiming: Evidence From a Multifractal Analysis With Comparisons to Linear Surrogates in the Fitts Task

**DOI:** 10.3389/fphys.2019.00998

**Published:** 2019-08-07

**Authors:** Christopher A. Bell, Nicole S. Carver, John A. Zbaracki, Damian G. Kelty-Stephen

**Affiliations:** ^1^Department of Psychology, Grinnell College, Grinnell, IA, United States; ^2^Department of Psychology, University of Cincinnati, Cincinnati, OH, United States

**Keywords:** motor coordination, Fitts task, visual perception, anticipation, multifractal

## Abstract

Movement coordination depends on directing our limbs to the right place and in the right time. Movement science can study this central requirement in the Fitts task that asks participants to touch each of two targets in alternation, as accurately and as fast as they can. The Fitts task is an experimental attempt to focus on how the movement system balances its attention to speed and to accuracy. This balance in the Fitts task exhibits a hierarchical organization according to which finer details (e.g., kinematics of single sweeps from one target to the other) change with relatively broader constraints of task parameters (e.g., distance between targets and width of targets). The present work seeks to test the hypothesis that this hierarchical organization of movement coordination reflects a multifractal tensegrity in which non-linear interactions across scale support stability. We collected movement series data during a easy variant of the Fitts task to apply just such a multifractal analysis with surrogate comparison to allow clearer test of non-linear interactions across scale. Furthermore, we test the role of visual feedback both in potential and in fact, i.e., by manipulating both whether experimenters instructed participants that they might potentially have to close their eyes during the task and whether participants actually closed their eyes halfway through the task. We predict that (1) non-linear interactions across scales in hand movement series will produce variability that will actually stabilize aiming in the Fitts task, reducing standard deviation of target contacts; (2) non-linear interactions across scales in head sway will stabilize aiming following the actual closing eyes; and (3) non-linear interactions across scales in head sway and in hand movements will interact to support stabilizing effects of expectation about closing eyes. In sum, this work attempts to make the case that the multifractal-tensegrity hypothesis supports more accurate aiming behavior in the Fitts task.

## Introduction

One of the central and fundamental capacities of the typically developing human movement system is to extend a limb toward a target. Very much of movement coordination is the timely movement of one limb from one target to the next. So, it is no mistake that the Fitts (1954) task remains a central paradigm of movement-science research: the Fitts task asks participants to make contact with one of two targets in alternation, swiftly and accurately, and in so doing, it epitomizes the challenge of movement coordination. The primary goal of this manuscript is to examine this task in terms of hand contacts with the target. This manuscript is not an attempt to document or explain Fitts law as in Meyer et al. (1988). We use the Fitts task in a low-difficulty setting merely to measure a simple, low-stakes case of goal-directed behavior.

In this low-stakes adaptation of the Fitts task, we hope to show five points of evidence about the relatively unconstrained use of vision for planning tactile interaction with a task environment over time. First, this simple task of making manual contact with targets in alternation depends on non-linear interactions over time across the body. Second, we know the first point because multiple parts of the body generate multifractal fluctuations during this task. Third, across progressively more cycles of alternation, stronger multifractal evidence of non-linearity at the hand predicts greater stability (i.e., lower standard deviation) in contact with targets. Fourth, again, across progressively more cycles of alternation, stronger multifractal evidence of non-linearity at the head supports use of visual information for making more stable contact with the targets – both the use of current visual information with eyes open and the use of past visual information after closing the eyes. Fifth and lastly, anticipating a loss of visual information stabilizes contacts with the targets, but this stabilizing effect of anticipation depends on the mixture multifractal evidence of non-linearity both at the head and the hand.

A key aspect of movement coordination that the Fitts task epitomizes is hierarchical organization. The Fitts task requires a long run of movements through repeated cycles. The Fitts law relates movement times with a difficulty index defined as between-target distance divided by target width (Fitts, 1954). So, the Fitts task prompts variations of movement amplitude between targets and variations of accuracy within targets. Within this long run of cyclical movements, individual cycles show a finer pattern of accelerations and decelerations. As the movement system lands its limb on one target or the other, it makes use of varying degrees of correction. Besides the obvious constraints of the difficulty index on motor kinematics, Wijnants et al. (2012) showed in a manual Fitts task that the parameters of the task changed how stability in target contacts spread across time, from one cycle to the next. The evidence for their claim was a variety of fractal fluctuations, with different degrees of fractal structure under different task settings. Hence, even the simple demands of the Fitts task show how global constraints can produce changes in finer-scaled movement dynamics. A fractally scaled measurement is one that exhibits a scale-invariant decay of variability, with short- or small-scale behaviors showing close correlation with the longer-range or more global constraints of a task. So, multiple fractal results from measured movement performance offer a glimpse the movement system’s hierarchy of cross-scale interactions.

This hierarchical form and the consonant fractal geometry have rich potential for supporting the movement system’s solution of vast challenges in movement coordination. The multifractal tensegrity hypothesis makes hierarchically organized interactions central to the explanation of dexterous behavior. Specifically, this perspective suggests that the interaction across scales is the hallmark of dexterity. It appeals to the physiological evidence that the human movement system embodies a balance between tensions and compressions across many nested scales, e.g., cellular, tissue, organ, synergy, and organism levels of analysis (Turvey and Fonseca, 2014). Outside of this perspective, evidence of multifractal fluctuations in various physiological or behavioral measures often appears only as an exotic by-product in the residual term of mechanistic or anatomical models of the movement system (Chen et al., 1997; Slifkin and Eder, 2012). That is to say, theories of movement that emphasize anatomical motor components more than softly-assembled interactions might recognize the multifractal evidence, but they might consider it only “noise” independent from the underlying mechanism or as an artifact of the underlying mechanism.

The multifractal tensegrity hypothesis takes a different view and makes multifractal geometry part of the central hypotheses about what drives motor coordination. It begins from the premise of expecting an interaction-driven architecture across all scales of the movement system, and it takes the expectation of multifractal structure as a logical prediction. According to this interaction-driven architecture, the aforementioned tension-compression balance reflects an architectural/morphological principle of “tensegrity.” The term “tensegrity” is a neologism short for “tensional integrity.” This tensional integrity certainly permits the distinction of anatomically distinct components. However, this tensional integrity entails that components hang together in a tightly-knit network relationship that allows local perturbations to spread globally. Thus, tensegrity allows global configuration to absorb and constrain minor local perturbation and to translate larger local perturbations into new global stabilities (Ingber, 2006). A major entailment of tensegrity principles is fractal and, more generally, multifractal geometry. The nested tension-compression balance generates fractal shapes in a purely symmetric or unbounded system (e.g., nested icosahedra extending in all directions). Without any bounds or constraints on this abstract requirement for balancing tension with compression, there is no pressure for any deviation from the same power-law to govern the entire system. However, as soon as we embed this system into a context or add any constraints, the constraints produce local deviations from the originally abstract uniformity. These constraint-driven local deviations press the tensegrity system into embodying a variety of power-law forms in which the deviations from the original requirement to balance tensions and compressions manifest in deviations from a single power-law description throughout. Put more simply, the deviations produced by constraints produce deviations in how cross-scale interactions generate the power-law relations. The constraint of any asymmetries or finite bounds (e.g., the skin surrounding the movement system or the size and shape of a task environment) will turn this originally fractal tensegrity into a multifractal form (Gouyet, 1996; Skelton and de Oliveira, 2009). Crucially, the heart of the multifractal tensegrity hypothesis is that estimates of multifractal evidence should speak directly to the form of multi-scaled tension-and-compression balance (e.g., Mandelbrot, 1974; Schertzer and Lovejoy, 1985; Halsey et al., 1986).

The multifractal tensegrity hypothesis has shown early promise across a wide range of scientific programs. First, there has been broad evidence that human physiology exhibits multifractal fluctuations suggesting cascade-driven dynamics (Ivanov et al., 1999, 2001, 2002, 2004; Amaral et al., 2001). Second, the formal structure of tensegrity principles has ready use in in biomedical engineering projects alongside the explicit intention to design structures with the right morphology (e.g., again, nested icosahedra) to mesh with existing physiology of a movement system (e.g., Chambliss et al., 2013). Third, and more to the present work’s point, the statistical-geometrical signature of multifractal geometry allows us to recognize those formal structures in the biological, behavioral “wild.” The present work features tensegrity as a reason to expect that multifractal patterning of movement variability is going to be a powerful predictor of dexterous behaviors (Kelty-Stephen, 2018). What follows below will take up multifractal geometry almost to the exclusion of using the word “tensegrity,” but we plant the multifractal-tensegrity hypothesis flag here at the outset to make clear that the tensegrity principles at play in the movement system implicate multifractal geometry as a privileged window on the tensegrity principles supporting dexterous behavior, specifically for this article, in the Fitts task. In this multifractal-tensegrity view, dexterous behavior assembles itself at any constituent level of this movement system as a consequence of explicitly non-linear cross-scale interactions. What Wijnants et al. (2012) found regarding the Fitts task aligned with this perspective, but it remains to actually test that non-linear interactions across scale are actually good for stabilizing task performance.

The present work aims to tailor empirical Fitts-task research to clarify specifically the relevance of the multifractal-tensegrity perspective. A general prediction that we hope to support is that the non-linear interactions across scale in the movement system support more accurate, more stable, and less variable achievement of the task. We use measured time series of movement variability and submit them to multifractal analysis to assess these non-linear interactions across scale. The idea that movement variability can promote greater stability might initially seem counterintuitive. However, it grows from the two sequential points: first, that movement variability is not equivalent to error and second, that movement variability might sooner afford ongoing exploration and corrections to ongoing sensory and kinesthetic information. That is, movement variability serves to enrich the movement-system’s relationships with the task environment. Hence, by enriching this relationship, more movement variability can stabilize performance (Stoffregen, 1985, 1986; Stoffregen and Riccio, 1990; Riccio, 1993; Stoffregen et al., 2005; Stergiou and Decker, 2011; Sternad, 2018). The proposed tensegrity architecture of the movement system suggests that multifractal estimates should best portray the variability expected to promote stability. We will quantify the movement system at the head and at the hand as it moves the hand between targets in alternation from target to target over 10 min blocks. We expect that the statistical multifractal signatures of non-linearity estimated from measurement movement will predict more focused, less variable hand positions at target contacts. Testing this prediction will involve two changes: one data-analysis change and one methodological change.

The data-analysis change will be the use of multifractal analysis in concert with surrogate testing. Using multifractal analysis to quantify the strength of non-linear cross-scale interactions requires comparison with surrogate-data time series (Ihlen and Vereijken, 2010). We expect that non-linear interactions across scales should support and indeed stabilize aiming and so reduce aiming variability in the contacts with the Fitts-task targets. Given the tensegrity structure of the movement system, multifractal geometry should have a privileged ability to quantify this variability. For instance, we know that variety of power-law scaling of trial-by-trial movement variability supports more accurate trial-by-trial use of perceptual information (Stephen et al., 2010; Stephen and Hajnal, 2011; Kelty-Stephen and Dixon, 2014). The difference here is that we can actually test whether this non-linear cross-scale interaction explicitly predicts this accuracy by testing whether the standardized marginal difference between “multifractality of original series” and “multifractality of surrogate series” predicts less aiming variability and so greater precision in the Fitts task. So, what we are implicating is, literally, a *t*-statistic: “multifractality of original series” minus “multifractality of surrogate series” divided by the standard error of surrogate multifractality. We will call this *t*-statistic *t*_MF_ for short to abbreviate “*t* statistic for multifractal difference between original series and surrogate data.” The linear standard represented by the surrogate merely allows us to estimate multifractal structure as if there were no non-linear interactions. We aim to make the very specific prediction that standard deviation in target contacts in a Fitts task varies inversely with non-linearity *t*_MF_.

The methodological change is to have participants actually close their eyes in the middle of the task (i.e., between two equal-length blocks of the task) and even to give participants the expectation at the outset of potentially having to close their eyes. This change is to give force to speculation elsewhere about the role of visual feedback. Specifically, this manipulation would interrupt any visual information that might operate as an independent component exerting mechanistic effects producing non-fractal fluctuations (e.g., Slifkin and Eder, 2014). However, through the perspective of expecting a multifractal tensegrity, the manipulation of having eyes close during the task would further the interactions across scale. Specifically, under these conditions, vision serves two roles: first, at a short time scale, using current visual information for current behavior and, second, over a longer time scale, using visual information to anticipate the potential loss of visual information. This work would build on previous research that has shown that multifractality predicts the use of current visual information as well as the use of past visual information with eyes closed, both through sequential monofractal analyses (Stephen et al., 2010; Stephen and Hajnal, 2011; Kelty-Stephen and Dixon, 2014) as well as through *t*-statistic *t*_MF_ comparing series multifractality to surrogate multifractality (i.e., non-linear interaction across scales) in head sway (Carver et al., 2017). The present work tests whether multifractal non-linearity in head-sway can moderate the short-scale effect of closing eyes and moderate the longer-range effect of expectation.

Interactions across time scales should mean that visual information available shortly before closing eyes may persist longer in participants with greater multifractal non-linearity of head sway, and so although closing eyes should result in greater aiming variability, multifractal non-linearity of head sway may reduce aiming variability following the actual closing of eyes. Previous research into anticipation has already indicated relevance of multifractal evidence of non-linear interactions in supporting coordination of several events into an organized, anticipatory response under uncertainty (Stephen and Dixon, 2011; Marmelat and Delignieres, 2012; Torre et al., 2013; Delignieres and Marmelat, 2014; Almurad et al., 2017a,b). Hence, we expect to leverage the known possibility that multifractality of bodily movement supports anticipation. Giving participants the expectation of potentially closing eyes at the outset might prompt an anticipatory mixing of visual information with haptic information about hand behavior. So, we expect that the interaction of *t*_MF_ at head and hand should moderate the effect of expectation in reducing aiming variability.

The present work situated the Fitts task firmly in the foundation of multifractal tensegrity and tested whether the strength of multifractal signatures of non-linear cross-scale interactions actually support greater accuracy and so less aiming variability. We tested three hypothesized effects that multifractal-indicated non-linearity *t*_MF_ will have in stabilizing accuracy (i.e., diminishing variability in the hand’s target contacts with each cycle) in the Fitts tasks: (1) greater *t*_MF_ in the hand predicting greater accuracy (lower standard deviation) in general, (2) greater *t*_MF_ at the head predicting greater accuracy (lower standard deviation) following the closing of eyes, and (3) the interaction of greater *t*_MF_ of hand and of head predicting greater aiming accuracy under the expectation of closing one’s eyes.

## Materials and Methods

### Participants

Twelve Grinnell College students participated in this experiment after providing informed consent according to the guidelines of the Grinnell College Institutional Review Board and in accordance with the Declaration of Helsinki. All participants were right-handed, had normal or corrected-to-normal vision, and exhibited no motor impairment. The motion-capture suits malfunctioned for the second half of one participant’s task, making the data for that one half of one participant’s data unusable. Data analysis included this participant’s first half of the task but excluded this participant’s second half.

### Apparatus

Participants sat at a standard office desk. Two wooden blocks (1.5^″^ on each side) were glued to the desktop 10 inches (25.4 cm) away from one another and within comfortable arm’s reach. A Noitom Perception Neuron motion-capture suit recorded movements throughout the entire experiment and logged the data at 60 Hz using Noitom’s companion Axis Neuron software.

### Design

There was a 2 (Expectation) × 2 (Eyes-Closed in Block 2) × 2 (Block) mixed design. All participants completed two 10 min blocks of the task. Each participant was randomly assigned to either level of Expectation (i.e., either “Expecting that the experimenter might ask them to close their eyes later on” or “No expectation of the experimenter asking them to close their eyes later on”). Each participant was also randomly assigned to either level of Eyes Closed in Block 2 (i.e., “Closing their eyes during the Block 2” or “Keeping eyes open during Block 2”). Three participants appeared in each of the four between-groups crossings of Expectation with Eyes-Closed in Block 2.

### Procedure

Experimenters helped participants put on the motion capture suit and then instructed them to sit at the desk and explained that the task would be to use the fingers of their dominant hand to touch the each of the blocks attached to the desk in alternation as fast and regularly as possible. Participants in one level of the Expectation independent variable heard the experimenter instruct them that they might have to close their eyes at some undetermined point during the task and to continue completing the fast, regular contact with the target. Participants in the other level of the Expectation independent variable heard no such instructions. All participants completed the task with their eyes open for the first 10 min block. After 10 min of the task, experimenters asked participants in one level of the Eyes-Closed independent variable to close their eyes for the remainder of the task, but experimenters made no such request to the participants in the other level of this independent variable, allowing them to continue the task with eyes open. Participants completed the next 10 min of the task with either eyes open or eyes closed. There was no break between Block 1 and Block 2 except to recalibrate the machine.

The motion-capture suit collected upper-body position information about head position and dominant-hand position.

### Analysis

We computed multifractal analysis and surrogate testing on non-overlapping epochs of the continuous lateral position series for the dominant hand as it moved alternately from left to right. By “lateral position,” we mean the position of the hand relative to the midpoint between the targets, parallel to a line connecting the targets. As for the head movement series, we reduced the three-dimensional position series of the head to interpoint Euclidean-distance series indicating the Euclidean distance between each pair of consecutive 3-dimensional positions (e.g., Kelty-Stephen and Dixon, 2014). Whereas task constraints and the definition of the outcome variable made it effective to focus strictly on only one dimension of the hand position, we aimed to collapse as much of the multidimensional structure of the head variability into a similarly one-dimensional time series. We computed multifractal analysis and surrogate testing on the same non-overlapping epochs of interpoint Euclidean distance series indicating head movements as we used for the hand-movement series. Use of the same non-overlapping epochs allowed us to align the concurrent multifractal estimates from hand and head. We compared multifractal estimates to the outcome variable of manual-aiming variability in this lateral direction, which outcome we abbreviate below as SD(Aim) to indicate that we are using regression modeling to predict how standard deviation of manual contacts with the target changes within epoch. We defined epochs not in terms of raw time but in terms of how many cycles the participant produced. We defined epochs of three size: 20 cycles, 40 cycles, and 80 cycles. This approach allowed us to evaluate effects on the task outcome of variability on the grounds of an equal number of cycles and/or manual contacts with the target (i.e., two manual contacts per cycle) across time and across participant. Having no prior intuitions on which to lock epoch size to one specific value and wanting explicitly to examine the relationship between multifractal structure and adaptation to task constraints across time, we tested this relationship by using three separate epoch sizes, thereby generating three sets of multifractal estimates and three corresponding sets of SD(Aim) outcome estimates. We compiled all results into one regression model to reveal whether effects of multifractal structure might be brief or long-term (e.g., Franca et al., 2018).

#### Multifractal Analysis in General

In a broad sense, newcomers to multifractal analysis might understand multifractality as a variability measure where we may not necessarily assume the homogeneity that makes a standard deviation so effective for quantifying variability. Linear modeling can use the linear autocorrelation (or, equivalently, the Fourier-amplitude spectrum) to model temporal sequence. However, non-linear time series may exhibit not just one autocorrelation but several – whether changing with time (i.e., changes in autocorrelation function with time) or changing according to fluctuation size (i.e., differently large or small fluctuations have different autocorrelations). Multifractal analysis covers a broad class of ways to query the variation of temporal sequence, and we apply it to both the original series and to surrogate series that have the same values and the same linear autocorrelation. The contrast of original-series multifractality and surrogate-series multifractality allows comparing the multifractal spectrum width in both cases and using the difference between them as a measure of non-linear interactions across time scale.

#### Multifractal Analysis by the Chhabra and Jensen Method (CJ)

We use the Chhabra and Jensen (1989) method to estimate the multifractal spectrum width because it is not sensitive to sinusoidal trends free to vary across time – while finite-variance scaling methods are (Bashan et al., 2008; Kelty-Stephen et al., 2013). We preferred the direct algorithm to the wavelet transform modulus maxima method as well (Muzy et al., 1991, 1993, 1994) which is based in the same reliance on finite variance (Eke et al., 2002) and shows strong agreement with multifractal detrended fluctuation analysis, both in its resulting estimates and in its sensitivity to quasiperiodic trends (Zhou et al., 2013).

**FIGURE 1:**
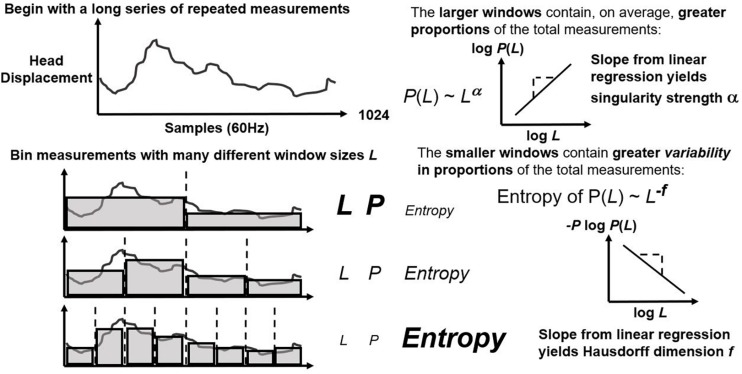
Schematic of first steps of multifractal algorithm that bin a repeated measure on many scales (left panel) and that estimate linear relationships between logarithmic average proportion and bin size as well as between Shannon entropy and bin size.

**FIGURE 2:**
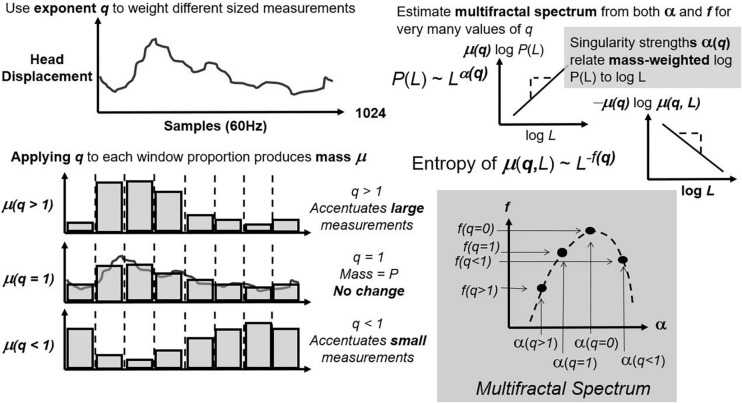
Concluding steps of multifractal analysis that use parameter *q* to accentuate proportions of different size **(left panel)** and then re-estimate the same exponents as in Figure 1 for each new value of q **(bottom panels)**. The multifractal spectrum comprises ordered pairs of exponents for matching q.

First, CJ partitions (or “bins”) the series over sequences with lengths varying from four points to a fourth of series length (Figure 1; left panel). It estimates exponents α and *f* as the relationships of average bin proportion and Shannon entropy (Shannon, 1948; Figure 1; right panel), respectively, with bin size. A mass μ estimate weight proportions according to proportion size (see Figure 2, left panel). *q*-based variability with mass μ(*q*) generalizes single α and *f* estimates from earlier steps into continua α(*q*) and *f*(*q*). Multifractal analysis distinguishes temporally heterogeneous series from homogenous series by the amount of variety in α(*q*) and *f*(*q*) for all values of *q* yielding stable power-law relationships. The multifractal spectrum is the set of ordered pairs (α(*q*), *f*(*q*)), an often-asymmetric and inverted U-shaped relationship (Figure 2, right panel). We included α(*q*) and *f*(*q*) whenever log-scaled linear fits correlated at *r* of 0.995 for the log-scaled linear fit. We excluded values of *q* either (1) for which mass-weighted proportion and for which Shannon entropy were undefined (e.g., due to masses rounding to zero) or (2) power-law fits for which *r* < 0.995. The final output of multifractal analysis here is the multifractal spectrum width *w*_MF_ equal to the maximum estimated α(*q*) minus the minimum estimated α(*q*).

#### Surrogate Comparison

**FIGURE 3:**
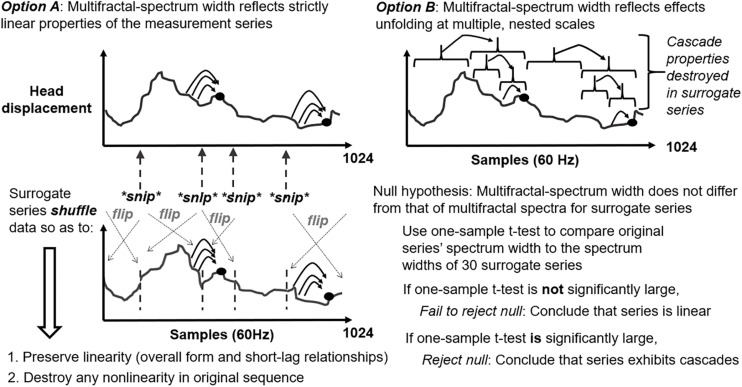
Schematic of iterative amplitude adjusted Fourier transform (IAAFT) surrogate-generation procedure for representing the null hypothesis of linearity **(left panel)** along with schematic articulation of the alternative hypothesis of non-linearity **(right panel)**.

Multifractal-spectrum width varies with non-linear interactions across time scales but also with histogram skew or linear autocorrelation. Hence, evidence of non-linear interactions requires comparing multifractal-spectrum width for original series to multifractal-spectrum widths for a sample of (here, 30, following the precedent of Ihlen and Vereijken, 2010) surrogate series that re-order original series’ values while preserving linear autocorrelation and destroying original sequence (i.e., iterative amplitude-adjusted Fourier-transform; IAAFT; Schreiber and Schmitz, 1996; Ihlen and Vereijken, 2010; schematized in Figure 3). Generating IAAFT surrogates involves taking the Fourier transform of the repeated-measures series and repeatedly scrambling phase spectrum while preserving the amplitude spectrum. As noted above, the *t*-statistic *t*_MF_ encoded the standardized difference in multifractal-spectrum width *W*_MF_ between original measured series and average multifractal-spectrum width *W*_MF_ across 30 surrogate-data series, divided by standard error of surrogate-data series’*W*_*MF*_. That is *t*_*MF*_ = (*W*_*MF*_ of original series – Average *W*_*MF*_ of surrogate-data series)/(Standard error of W_MF_ of surrogate-data series). *T*_MF_ indicates non-linear interactions across time scales in a standardized way that should generalize across series with differing histogram skew or linear autocorrelation.

These *t* statistics reflect the comparison of each original fluctuation series’ multifractal spectrum to a sample of multifractal spectra generated from each of 30 phase-randomized surrogates preserving the full linear description (i.e., mean, standard deviation, and autocorrelation) of the original fluctuation series. Any difference between original multifractal spectrum width and average surrogate multifractal-spectrum width must reflect a non-linearity – particularly if it falls outside of the 95% confidence interval, yielding a significant *t* statistic. Because the phase-randomized surrogates already include the autocorrelation over all time scales independently, this term “non-linearity” is specifically equivalent with the phrase “non-linear interactions across time scale.”

#### Multiple Linear Regression Model Testing Effects of Multifractal-Indicated Non-linearity

A single mixed-effect linear regression (e.g., Singer and Willett, 2003) using R library “nlme” (Pinheiro et al., 2018) modeled the dependent variable of “standard deviation of hand position relative to target position during target contact” [i.e., SD(Aim)] as the sum of the following interactions as well as all component lower-order interactions and main effects: Expectation × Number(Epoch) × Size(Epoch) × *t*_*MF*_(Hand) × *t*_*MF*_(Head), Closedeyes × (Number[Epoch] + Size[Epoch] + Expectation) × *t*_*MF*_(Head), Expectation × Number(Epoch) × *W*_*MF*_(Hand) × *W*_*MF*_(Head), Closedeyes × (Number[Epoch] + Size[Epoch] + Expectation) × (Mean[Head] + SD[Head]), and Number(Epoch) × Half.

The first two sets of interactions tested the explicit hypotheses noted above. The second two sets of interactions controlled for simpler effects that a more skeptical point of view could want represented. We detail both in the next two subsections.

##### Interactions addressing the explicit hypotheses

The first two sets of interactions tested the explicit hypotheses. Specifically, the higher-order interaction Expectation × Number(Epoch) × Size(Epoch) × *t*_*MF*_(Hand) × *t*_*MF*_(Head) and its component lower-order interactions and main effects tested whether the ongoing effect of expectation on SD(Aim) depended on epoch size as well as multifractal-indicated non-linearities in hand and head positions. In plainer language, this set of terms poses the questions: “Do non-linear interactions across time in the hand stabilize aiming? (Hypothesis 1) Does that support work with hand non-linearity to stabilize aiming under the anticipated threat of potentially losing visual information?” (Hypothesis 3).

The higher-order interaction Closedeyes × [Number (Epoch) + Size(Epoch) + Expectation] × *t*_*MF*_(Head) and its component lower-order interactions and main effects tested whether the ongoing effect of closed eyes on SD(Aim) depended on multifractal-indicated non-linearity in head position with moderation by expectation as well as by the size and number of epoch used to quantify standard deviation for SD(Aim). In plainer language, this set of terms poses the question: “Does the effect of closing one’s eyes on aiming variability depend on non-linear interactions across time at the head?” (Hypothesis 2).

##### Interactions addressing control effects and representing more skeptical views

The remaining interactions provided control effects to address various more skeptical questions worth asking about SD(Aim). That is, these lower-order effects give the regression model due opportunity to control for the simpler explanation. They represent the possibility that comparison to surrogates and change with epoch size are each needless complications. Specifically, the higher-order interaction Expectation × Number(Epoch) × *W*_*MF*_(Hand) × *W*_*MF*_(Head) and its component lower-order interactions and main effects tested whether the ongoing effects of expectation on SD(Aim) for any epoch size dependent not on non-linearity but just multifractal-spectrum width. That is to say, they answer the following question: “Surrogate testing sounds difficult, and non-linear interactions across time scale sounds like splitting of computational hairs. Shouldn’t multifractal analysis be enough to explain the contribution of hand and head sway to aiming variability?”

For that matter, from such a diligently skeptical point of view, we could doubt the usefulness of this multifractal mathematics at all. The next set of control terms offers a test representing this skeptical point of view. The higher-order-interaction Closedeyes × (Number[Epoch] + Size[Epoch] + Expectation) × (Mean[Head] + SD[Head]) and its component lower-order interactions and main effects tested whether any effect of closed eyes on SD(Aim) was sooner dependent on simpler descriptive statistics. That is to say, in plainer terms, “Shouldn’t aiming variability with eyes closed sooner depend on the average size and average variability of head sway? And shouldn’t these simpler statistics fewer algorithmic steps away from the raw data sooner show the relationships better with time, time span, and the threat of potentially losing visual information?”

The last set of control effects described by the interaction Number(Epoch) × Half and its component main effects offers the model a way to articulate the simple effect of time. The plain-language question posed by these terms would be “Won’t fluctuations in hand and head also simply vary across continued time in the task regardless of potential or action loss of visual information?”

## Results and Discussion

**FIGURE 4:**
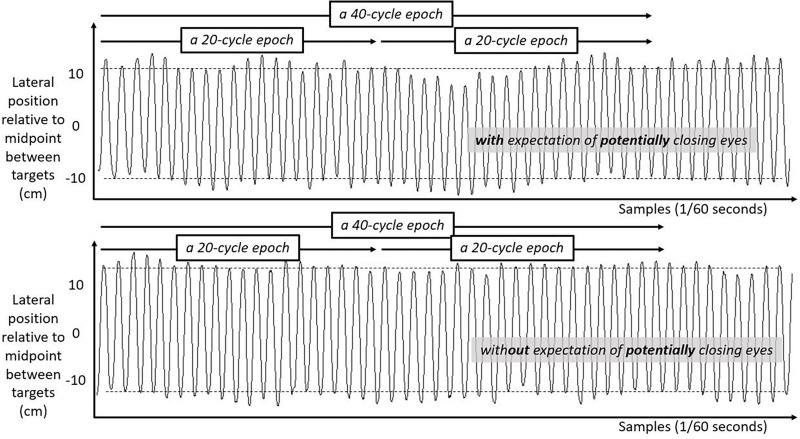
Lateral hand position over time for 50 s in the first half of the task. Solid lines show the lateral position of the hand over time in the first half of the task for two participants both with their eyes open. **Top panel** shows movement series for a participant expecting that they might have to close their eyes later. **Bottom panel** shows movement series for a participant with no expectation of having to close their eyes later. Dashed horizontal lines schematize the average lateral position of manual contacts from which aiming variability SD(Aim) was calculated.

**FIGURE 5:**
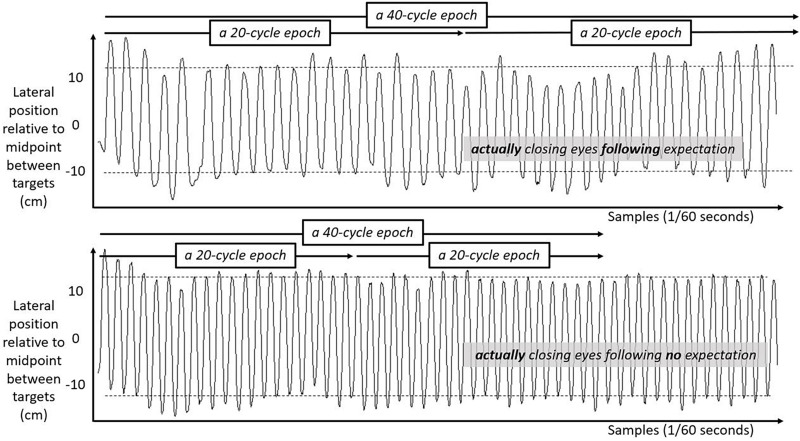
Lateral hand position over time for 50 s in the second half of the task with eyes closed. Solid lines show the lateral position of the hand over time in the second half of the task for two participants both with their eyes closed. **Top panel** shows movement series for a participant who had expected that they might have to close their eyes. **Bottom panel** shows movement series for a participant who had no expectation of having to close their eyes. Dashed horizontal lines schematize the average lateral position of manual contacts from which aiming variability SD(Aim) was calculated.

**FIGURE 6:**
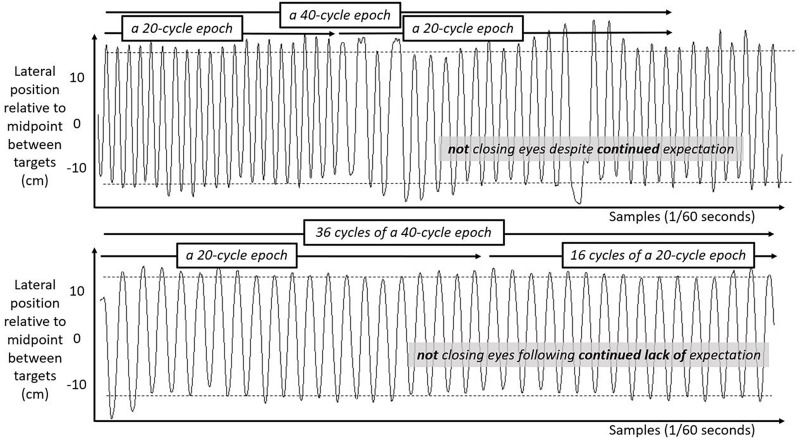
Lateral hand position over time for 50 s in the second half of the task with eyes open. Solid lines show the lateral position of the hand over time in the second half of the task for two participants both with their eyes open. **Top panel** shows movement series for a participant who had expected that they might have to close their eyes. **Bottom panel** shows movement series for a participant who had no expectation of having to close their eyes. Dashed horizontal lines schematize the average lateral position of manual contacts from which aiming variability SD(Aim) was calculated.

We include three figures to depict example movements of the hand during 50 s portions of the Fitts task under each case. Figure 4 shows the lateral position of the hand over time in the first half of the task. Figure 5 shows the lateral position of the hand over time in the second half of the task with eyes closed, both for a participant who expected they might have to close their eyes and for a participant who did not expect to close their eyes. Figure 6 shows the lateral position of the hand over time in the second half of the task with eyes remaining open, both for a participant who expected they might have to close their eyes and for a participant who did not expect to close their eyes. Again, in all cases, these figures depict 50 s in the task, extremely small subsets of the 10 min halves of the task.

**FIGURE 7:**
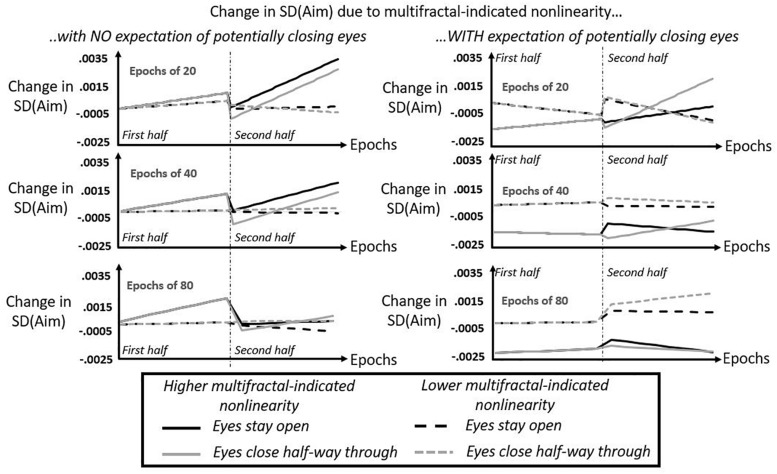
Model predictions from regression of SD(Aim) by expectation of potentially closing eyes, by epoch, by half of task, by *t*_*MF*_, and by eyes being closed or open. Solid lines show model-predicted contributions of multifractal-indicated non-linearity to aiming variability at the hand (SD[Aim]) for higher *t*-statistics *t*_*MF*_ (predictions shown specifically for 3rd quartile of t_MF_) for participants keeping their eyes open in the second half (black solid lines) and for participants closing their eyes for the duration of the second half (gray solid lines). Dashed lines show model-predicted contributions of multifractal-indicated non-linearity to aiming variability at the hand (SD[Aim]) for lower *t*-statistics *t*_*MF*_ (predictions shown specifically for 1st quartile of *t*_*MF*_) for participants keeping their eyes open in the second half (black solid lines) and for participants closing their eyes for the duration of the second half (gray solid lines). Dash-and-dotted vertical lines separates the first and second halves of the task, and it is noteworthy that gray and black lines are overlapping across the board in the first halves of the task. Left panels depict model predictions for participants without the expectation of potentially having to close their eyes, and right panels depict model prediction for participants with this expectation. Top, middle, and bottom panels depict model predictions for epochs containing 20, 40, and 80 cycles, respectively.

The regression model returned a number of lower-order significant effects on SD(Aim), all of which appear in Table 1. The resulting model predictions of epoch-by-epoch SD(Aim) correlated with actual epoch-by-epoch SD(Aim), *r* = 0.7335. The regression-model predictions appear in Figure 7. First, we review the multifractal-free results of this modeling. Next, we review the effects that multifractality had on SD(Aim).

**TABLE 1:** Significant effects from regression modeling of manual-aiming variability SD(Aim).

**Predictor**	**Coefficient**	**SE**	***p***
**EFFECTS OF MULTIFRACTALITY OF HAND DUE TO NON-LINEARITY, t_MF_(Hand)**			
Number(Epoch) × *t*_*MF*_(Hand)	1.17 × 10^–5^	5.56 × 10^–6^	<0.05
Number(Epoch) × Size(Epoch) × *t*_*MF*_(Hand)	−7.23 × 10^–7^	2.86 × 10^–7^	<0.05
**EFFECTS OF MULTIFRACTALITY OF HEAD DUE TO NON-LINEARITY, t_MF_(Head)**			
Closedeyes × *t*_*MF*_(Head)	−2.10 × 10^–4^	9.67 × 10^–5^	<0.05
Closedeyes × Number(Epoch) × *t*_*MF*_(Head)	1.52 × 10^–5^	4.79 × 10^–6^	<0.01
Closedeyes × Size(Epoch) × *t*_*MF*_(Head)	3.80 × 10^–6^	1.50 × 10^–6^	<0.05
**EFFECTS OF MULTIFRACTALITY OF HAND AND HEAD DUE TO NON-LINEARITY, t_MF_(Hand) × t_MF_(Head)**			
Number(Epoch) × Size(Epoch) × *t*_*MF*_(Hand) × *t*_*MF*_(Head)	6.11 × 10^–8^	2.48 × 10^–8^	<0.05
Expectation × Number(Epoch) × Size(Epoch) × *t*_*MF*_(Hand) × *t*_*MF*_(Head)	−1.09 × 10^–7^	4.32 × 10^–8^	<0.05

While it is regrettable that this preliminary experiment did not have more individual participants, the regression model met the heuristic benchmark of having more than 10 observations per predictor (Harrell et al., 1996; Babylak, 2004; Finlay, 2014). Furthermore, *post hoc* power analysis using bootstrapping to resample the dataset 1000 times found greater than 80% for all effects reported, as well as for 17 other predictors in the model that were non-significant. Hence, the repeated measures aspect of this design compensated for the small sample size. Raw data are available on request and online^1^.

### Results Pertaining to Effects Non-multifractal Indicated Non-linearity

Supplementary Table 1 lists the significant effects of non-multifractal effects. SD(Aim) increased across halves of the experiment and for longer spans of time and varied with descriptive statistics of head sway, but this increase diminished within halves especially over the course of longer epochs. Closing eyes and actually losing visual information in the second half of the task did in fact increase SD(Aim). The effect of closing eyes and actually losing visual information interacted with the effect of the mere threat of potentially having to close eyes and lose visual information. Closing eyes accentuated relationships of expectation with head sway and SD(Aim).

### There Were No Effects of Multifractal-Spectrum Width

The absence of effects of multifractal-spectrum width on SD(Aim) suggesting indeed that multifractal analysis alone is insufficient to explain the contribution of hand and head sway to aiming variability. These null effects would on their own entail a failure to support hypotheses that multifractal analysis provides any insight into aiming variability in the Fitts task. However, the insufficiency of multifractal-spectrum width to predict aiming variability in the Fitts task is completely mute on the specific point of non-linear interactions across scales. As noted above, the better statistical signature of non-linear cross-scale interactions is the difference of multifractal-spectrum width between measured series and surrogate-data series. However, this use of surrogate data to estimate the non-linearity of cross-scale interactions appears to be carry significant weight in predicting variability in the Fitts task. And it appears to overshadow any such predictive weight for multifractal-spectrum width without surrogate comparison.

### Effects of Multifractal-Indicated Non-linear Interactions Across Scale

This section reviews the contributions of t_MF_(Hand) and *t*_*MF*_(Head). All of the following effects reviewed below appear schematized together in Figure 7. An important difficulty in interpreting these effects is that there were no main effects of either *t*_*MF*_(Hand) or of *t*_*MF*_(Head). To ease this task of interpreting these higher-order interactions, Figure 7 shows the summing together of these average effects of non-linearity on SD(Aim).

#### Effects of Non-linearity in Hand Fluctuations *t*_*MF*_(Hand)

As noted in the previous section, there were no main effects of t_MF_(Hand). Instead, the regression model showed SD(Aim) increased with greater hand non-linearity and with continued time in the task (Number[Epoch] × *t*_*MF*_[Hand] *B* = 1.17 × 10^–5^, *SE* = 5.56 × 10^–6^). This positive relationship between multifractal non-linearity and SD(Aim) over time decreased for longer time spans (Number[Epoch] × Size[Epoch] × *t*_*MF*_[Hand], *B* = −7.23 × 10^–7^, *SE* = 2.86 × 10^–7^), suggesting that stronger multifractal non-linearity at the hand predicted progressively less variable aiming behavior especially over longer time spans.

#### Effects of Non-linearity in Head Fluctuations *t*_*MF*_(Head)

Just as for t_MF_(Hand), there were no main effects of t_MF_(Head). Head non-linearity moderated the effect of actually closing eyes and losing visual information. Whereas closing eyes had produced a main effect of increasing SD(Aim), greater head non-linearity when closing eyes predicts a small but significant reduction in this increase (Closedeyes × *t*_*MF*_[Head], *B* = −2.10 × 10^–4^, *SE* = 9.67 × 10^–5^). That is to say, head non-linearity initially stabilized aiming variability at the hand, suggesting that head non-linearity appears to support the persistence of past visual information. However, the next two significant effects show that, whatever head non-linearity can do to prolong the impact of past visual information during the closed-eyes half of the task, this effect is fleeting both in terms of how fast it vanishes over time and how small a time span we must use to estimate non-linearity to see the effect clearly. Small but positive interactions of this effect with continued time in the task (Closedeyes × Number[Epoch] × *t*_*MF*_[Head], *B* = 1.52 × 10^–5^, *SE* = 4.79 × 10^–6^) and longer time span for evaluating aiming behavior (Closedeyes × Size[Epoch] × *t*_*MF*_[Head], *B* = 3.80 × 10^–6^, *SE* = 1.50 × 10^–6^) both attenuate this stabilizing effect.

We can summarize these effects of head non-linearity in the following ways. First, the participants’ awareness of potentially losing visual information seems to recruit head non-linearity – rather than hand non-linearity – as a stabilizing factor on aiming behavior [i.e., lowering SD(Aim)]. Second, irrespective of participant expectations, head non-linearity exerts an independent, additional stabilizing effect, reducing SD(Aim) perhaps by prolonging past visual information once participants actually close their eyes and lose current visual information. This evidence is thus precarious, with the first effect only marginally significant and the second effect significant but fleeting. As the next section shows, expectation remains necessary to knit the stabilizing effects of hand and head non-linearities together into a more reliable stabilizing effect on aiming variability.

#### Effects of Multifractal Non-linearity in Both Hand and Head Fluctuations *t*_*MF*_(Hand) and *t*_*MF*_(Head)

There were only two significant interactions involving *t*_*MF*_(Hand) × *t*_*MF*_(Head). Over longer time in the task and over longer time spans, increases in both hand and head non-linearities predicted that SD(Aim) would increase (Number[Epoch] × Size[Epoch] × *t*_*MF*_[Hand] × *t*_*MF*_[Head], *B* = 6.11 × 10^–8^, *SE* = 2.48 × 10^–8^). However, expectation significantly reversed this effect. Specifically, over longer time in the task and over longer time spans, the mere threat of potentially losing visual information along increases in both hand and head non-linearity predicted that SD(Aim) would decrease (Expectation × Number[Epoch] × Size[Epoch] × *t*_*MF*_[Hand] × *t*_*MF*_[Head], *B* = −1.09 × 10^–7^, *SE* = 4.32 × 10^–8^). That is to say that expectation changed the perceptual-motor impact of any relationship of non-linearity at the hand and at the head. The capacity of expectation to reduce aiming variability depends on multifractal non-linearity at both the head and the hand.

#### Integrating the Effects of Non-linearity by Conditions of Expectation and Closing Eyes

##### The middle 50% of non-linearity estimates predicts almost the same range of the actual epoch-by-epoch SD(Aim)

Figure 7 depicts the sum of all non-linearity-driven effects on SD(Aim), specifically for the middle 50% of the actual values of *t*_*MF*_(Hand) and of *t*_*MF*_(Head), i.e., model predicted change in SD(Aim) for the first and third quartiles of each t_MF_ variable. In all cases, Figure 7 only depicts high t_MF_(Hand) and high *t*_*MF*_(Head) together (in solid lines) and low *t*_*MF*_(Hand) and low *t*_*MF*_(Head) together (in dashed lines). For simplicity’s sake, Figure 7 does not show predictions for mixed cases. The maximum and minimum values on the y-axes for all panel indicates that the sum total of the non-linear-driven effects range from −0.0025 to 0.0035, spanning a range of SD equal to (0.0035 – (−0.0025) =) 0.0060. So, these modeled results do look subtle. It is certainly true that the non-linearity-driven effects were a minority of the total significant effects, but it is worth noting that the 0.0060-SD model-predicted change in SD(Aim) for this middle 50% of non-linearity estimates is by no means a modest proportion of the actual change in observed SD(Aim). The first and third quartiles of actual epoch-by-epoch SD(Aim) are 0.0131 and 0.0194, respectively, yielding a range of SD equal to (0.0194–0.0131 =) 0.0063. Hence, this model-predicted range of SD(Aim) using the actual middle 50% of non-linearity estimates covers about (0.0060/0.0063 =) 95.24% of the actual middle 50% of epoch-by-epoch SD(Aim). So, the subtlety of the effects in terms of absolute values in Figure 7 belies the fact that, actually, this predicted range is a substantial portion of the variance of epoch-by-epoch SD(Aim).

##### Without expectation of potentially closing eyes, multifractal non-linearity of hand and head is destabilizing factor most of the time and only stabilizing with after eyes close

Figure 7 provides a high-level view of many different ways to articulate the question about how non-linearity influences aiming variability. It depicts these relationships between non-linearity and SD(Aim) across the first and second 10 min halves of the task, allowing us to see the effects of closing eyes half way through (for the gray lines) relative to keeping their eyes open. Also, Figure 7 depicts these effects through different epoch sizes, i.e., different time spans for evaluating SD(Aim) and the corresponding predictors for each epoch of different size. In the left column representing model predictions for participants who did not expect to potentially close their eyes, we can see that, generally speaking, higher values of t_MF_(Hand) and t_MF_(Head) predict greater variability in aiming. Without this expectation, less multifractal non-linearity appears to predict greater aiming stability [i.e., change in SD(Aim) closest to zero] than multifractal non-linearity. Multifractal non-linearity seems to produce ever greater variability in aiming as we examine progressively longer epochs in the first half of the task, and the destabilizing aspect of multifractal non-linearity in the second half of the task is clearest in shorter epochs.

The only benefit of more multifractal non-linearity without expectation is that it appeared to reduce aiming variability compared to less multifractal non-linearity immediately upon closing of eyes half way through the task. That is, in the left column of Figure 7, the solid gray line dips into the negative range of SD(Aim) below the dashed gray line. This reduction of variability appears stronger in the shorter epochs, but although the reduction of variability is much smaller in longer epochs as noted above, this view of the suggests that this subtle effect is much longer lasting over the longer run in longer epochs.

##### Expectation of potentially closing eyes, multifractal non-linearity of hand and head becomes a stabilizing factor

Across the board, multifractal non-linearity of hand and head becomes a stabilizing factor for participants expecting that they may potentially have to close their eyes. That is to say, the expectation of potentially losing visual information makes greater multifractal non-linearity a stabilizing factor for aiming and makes less multifractal non-linearity a destabilizing factor for aiming. The only instance in which multifractal non-linearity fails to serve stabilizing role in aiming behavior is roughly the last two thirds of the second half of the task when we consider epochs of 20 cycles. For all other cases, multifractal non-linearity does appear to interact with expectation to support more stable aiming, i.e., lower SD(Aim).

As in the case without expectation, this pattern of results does predict an immediate benefit of multifractal non-linearity when participants close their eyes. The 20-cycle epochs shows a dramatic decrease in aiming variability for high multifractal variability (i.e., the solid gray line) that is only shallower when considering longer epochs. Hence, we see the same sharp, short, and fleeting decrease in SD(Aim) that we saw before without expectation (left column of Figure 7), but the model predictions suggest that expectation extends this stabilizing effect throughout more of the task especially over longer epochs. When eyes stay open (i.e., solid black lines), multifractal non-linearity generally continues to stabilize posture. The differences of multifractal effects with eyes open from the eyes-closed case appears to be twofold: it appears to stabilize aiming less than it would immediately after closing eyes, but it appears to exhibit more of the sustained stabilizing factor.

## Conclusion

We tested the hypothesis that multifractal-indicated non-linearity would moderate effects of expectation of potentially closing eyes and effects of actually closing eyes during the Fitts task. Specifically, we expected the non-linear results with wider multifractal spectra than their corresponding surrogates would be associated with lower aiming variability immediately after closing eyes and under the expectation of potentially closing eyes. The results supported this hypothesis. Specifically, we found a short, fleeting reduction in aiming variability under cases of non-linearly wider-than-surrogate multifractal spectra. We found the strongest evidence of this reduction and its subsequent disappearance when evaluating multifractality and aiming variability over the shortest epochs. We also found a sustained reduction of aiming variability following from the interaction of expectation and non-linearly wider-than-surrogate multifractal spectra. Primarily, what these results mean is that the non-linearity of bodily movements during the Fitts task support stable aiming behavior.

We can phrase these results as mostly affirmative answers to the specific questions raised in the section detailing the regression modeling of SD(Aim) that we defined as “standard deviation of hand position relative to target position during target contact.” In answer to “Do non-linear interactions across time in hand stabilize aiming? (Hypothesis 1), we can now answer “Yes.” Greater multifractal non-linearity in the hand reduced aiming variability. This effect echoes older findings that the multifractal structure in hand fluctuations supports more accurate use of mechanical and visual information (Stephen et al., 2010; Stephen and Hajnal, 2011; Kelty-Stephen and Dixon, 2014).

These results replicate a previously known effect of head-sway non-linearity for supporting the use of visual information (Eddy and Kelty-Stephen, 2015; Carver et al., 2017), confirming earlier suggestions from strictly multifractal evidence just short of testing for non-linearity in head sway (Hajnal et al., 2018). This known effect appeared through the stabilizing effect of multifractal non-linearity once participants closed their eyes. As detailed in the regression model, Hypothesis 2 asked “Does the effect of closing one’s eyes on aiming variability depend on non-linear interactions across time at the head?” The answer is “Yes.” Multifractal non-linearity at the head reduces aiming variability immediately after closing eyes, but the evidence for this brief stabilizing effect is clearest at the shorter epochs. Whatever capacity head sway has to embody interactions across time scales, it appears that this interaction of time scales may serve to prolong the effects of what visual information was available before eyes closed.

Head-sway non-linearity and hand non-linearity together moderated the effect of merely expecting potentially closing eyes. In answer to the question “Does that support work with hand non-linearity to stabilize aiming under the anticipated threat of potentially losing visual information?” (Hypothesis 3), we can now answer “Yes.” With or without prompting participants to actually close their eyes, expectation serves to diminish the reductive effect of hand non-linearity on aiming variability and to recruit head-sway non-linearity for reducing aiming variability instead. Multifractal non-linearity has its strongest stabilizing effect at the hand for longer epochs. Likewise, multifractal non-linearity at head and hand helps to translate expectation into stabilizing effects on aiming that become stronger for longer epochs.

Head-sway fluctuations thus appear to carry double duty for reducing aiming variability, both in supporting the use of visual information and supporting the expectation that visual information might not be available later on. It its key to emphasize that expectation only showed interactions with head-sway alone and with both head-sway and hand. Regarding the interactions with head-sway alone, it is possible that this stabilizing effect of expectation involved a new or further use of visual information, i.e., expectation might engender more or different of the non-linear patterns of exploration of the optical array nesting the manual aiming task. Regarding the interactions with head-sway and hand fluctuations, it is also possible that expectation plays upon synergy-like relationships within the organism between head and hand.

Candidate physiological explanations for these results could include localized tissues or specific subsystems. Certainly, heightened activity by sympathetic nervous system can increase change covariation among disparate physiological systems (e.g., Garcia-Retortillo et al., 2019). Expectation might provoke stress, anxiety, and uncertainty. Similarly, the strong relationships between the visual and vestibular systems (Abekawa et al., 2018; Ruhl et al., 2018) could explain these effects. However, qualifications, interactions, and context-sensitivities quickly clutter this seeming simplicity. For instance, vestibular system influences the sympathetic nervous system (Yates, 1992), suggesting that the alternatives are not mutually exclusive. Further, visual-vestibular interactions require qualification as well by aging (Lui et al., 2019), migraines (Bednarczuk et al., 2019), neuritis (Roberts et al., 2018), and interactions with somatosensation (Moro and Harris, 2018).

The present results speak directly to a physiological explanation residing in geometrical forms embodied by the physiological mechanism of connective-tissue networks weaving muscle, bone, nervous system together through the fascia. It allows multifractal estimation of non-linear interactions across scale and charting out the field dynamics of how this non-linearity ebbs and flows across an organism’s spatial or temporal extend (Chester et al., 2007; Dixon and Kelty-Stephen, 2012; Kelty-Stephen and Dixon, 2014; Carver et al., 2017). Indeed, broad interdependence of disparate physiological tissues is a central theme of the multifractal tensegrity hypothesis and a main motivation for using multifractal geometry to make explicitly estimable the non-linear interactions governing a cohesive system across many scales (Ingber, 2006; Van Orden et al., 2012; Turvey and Fonseca, 2014). Indeed, the dawning picture we have of the nervous system suggests a causal relationship run by non-linear interactions across scale (Morgane et al., 2005; Changizi and DeStefano, 2009; Deco et al., 2011). To the point, beyond muddying our ability to identify clear parts with reliably stable and distinct responsibilities, these non-linear interactions appear to be the very physiological support that allows the nervous system to operate as impressively as it does. Non-linear interactions across scale typify all physiological tissues and not just as a neat epiphenomenon of supposedly more real, more legitimate mechanism in local tissue. The multifractal tensegrity hypothesis acknowledges the multifractal-tensegrity aspect of physiological tissues as firmer ground for physiological explanation than local anatomical tissue.

The connective-tissue networks ground perception and action upon a wider-than-neural set of physiological tissues. Evidence of tensegrity support for perception and action includes contextually-specific responses proceeding faster than neural transmission alone would support – variously called “ultrafast,” “mechanotransduction” (to indicate the procession of mechanical rather than chemical causal forces), or, to distinguish from the slow reflex meandering across multiple synapses, “preflexes” (Brown and Loeb, 2000). These ultrafast, mechanically grounded responses appear across a wide spectrum of behaviors, from the activity of individual cells – both composing larger organisms (Ingber, 2006) or autonomous amoeba (Grebecka et al., 1999 – to responsivity of insects (Frantsevich and Gorb, 2002; Endlein and Federle, 2013) to humans locomoting (Moritz and Farley, 2004; Kiely and Collins, 2016), simply standing quietly (Marsden et al., 1983) or using language (Kelso et al., 1984; Moreno et al., 2011). Crucially, the ultrafast mechanotransduction to be explained is more generic than the local anatomical structures: e.g., amoeba and insects show sensitivity to mechanical rotation despite not having any clear analog to the vestibular system (Brunet, 1951; Hengstenberg, 1993; Svidersky and Plotnikova, 2002; Dilao and Hauser, 2013). So, tensegrity provides a rich background of tensions and compressions that precede, extend beyond, and directly modulate whatever the nervous system might do for a relatively narrow sliver of living behavior. Interactions and context-sensitivities amongst different neural subsystems are likely due to the tensegrity architecture pervading physiological tissues (Ingber, 2006; Turvey and Fonseca, 2014).

### The Non-linear Effects on Aiming Variability

These effects of multifractal-indicated non-linearity are not so exhaustive as to preclude effects of simpler statistics from head-sway. The method section’s detailing of the regression model notes that the inclusion of control effects allows us to ask the following: “Shouldn’t aiming variability with eyes closed sooner depend on the average size and average variability of head sway? And shouldn’t these simpler statistics fewer algorithmic steps away from the raw data sooner show the relationships better with time, time span, and the threat of potentially losing visual information?” Despite mean and standard deviation of head-sway registering significant main effects and interactions (Supplementary Table 1), none of these significant effects precluded evidence of multifractal non-linearity having the predicted interaction with closing eyes.

### No Effects of Multifractality That Are Not Due to Non-linearity

Multifractality appears worthless in this study except insofar as surrogate testing and resulting *t*-statistics produce multifractal-based estimates of non-linear interactions across scale. For all of these effects of multifractal-indicated non-linearity, notably absent were any effects of multifractal-spectrum width itself. As noted in the method subsection detailing the regression model, our modeling included a full set of interactions between expectation, epoch size, epoch number, and – where other interactions included the surrogate-based t-statistic – multifractal-spectrum width. We offered the interactions to represent the following sentiment and curiosity: “Surrogate testing sounds difficult, and non-linear interactions across time scale sounds like splitting of computational hairs. Shouldn’t multifractal analysis be enough to explain the contribution of hand and head sway to aiming variability?” Our model has entirely failed to find any support for this notion. Other variants of the Fitts task with greater difficulty may change this result, but for our present purposes, we meant only to test whether non-linearity was important for a very simple case of goal-directed behavior. Simple tasks elicit non-linearly complex behaviors, which point we do not see as a shortcoming or challenge to interpretation but rather as a signature of how pervasive non-linear interactions across scale can be.

Pervasiveness has been a key theme of earlier fractality-inspired discourse (e.g., Kello, 2011; Moscoso del Prado Martin, 2011). Here, we are keen to assign pervasiveness specifically to the non-linearity of interactions across scale entailed by multifractal results through surrogate comparison and not to the multifractality in itself, unqualified by surrogate comparison. Linear interpretations of multifractality certainly exist (i.e., unsystematic variation in the rate of divergence for strictly linear autocorrelation), but they reflect an attempt to fit the data that sooner exaggerates what is statistical articulable for mechanistic hypotheses than represents a logically plausible hypothesis (Gilden, 2009; Kelty-Stephen and Wallot, 2017). Indicating non-linearity of interactions across scale is the most substantive value that multifractality can have to inform complex-systems approaches to perception and action, and so, the present results of no multifractal effects that are not non-linear makes this demonstration particularly neat and uncluttered by linear counter-explanations of what multifractality means for perception and action. To the point, multifractal geometry is useful insofar as it quantifies the change in these pervasively non-linear interactions across scale.

### Implications

The present work sought to revisit a foundational task in movement science. Previous work applying fractal methodology to the Fitts task has focused on changes in fractal scaling in the movement-time or movement-amplitude series. In more difficult variants of the Fitts task, greater speed or greater accuracy leads to stronger evidence of fractal scaling in movement-time series or in movement-amplitude series, respectively (Wijnants et al., 2012). On the other hand, other research has sought to explain weakening of the fractal-scaling in movement-amplitude series due to a stronger use of visual feedback implicated by higher difficulty level (Slifkin and Eder, 2014). The present work attempts to delve more deeply into the movement variability at a finer grain, using more continuous time series, using multifractal rather than monofractal modeling, directly manipulating visual feedback rather than implicitly through difficulty level, and investigating the role of non-linear interactions across scales in hand and head to support the use of visual feedback in the Fitts task.

This work still observes position variability in aiming per cycle [i.e., SD(Aim)] as a key dependent measure. However, whereas movement-time and movement-amplitude series unfold at the same cycle-by-cycle scale of analysis, the present approach of analyzing fluctuations in the movement series per time sample drops below that cycle scale of analysis in order to investigate what more continuous structure may support the coarser cycle-by-cycle performance. Furthermore, in answer to the claims that temporal structure follows from visual feedback (Slifkin and Eder, 2014), this study experimentally manipulates visual feedback both in terms of participants’ expectations of potentially closing their eyes and in terms of participants actually closing their eyes. Also, whereas the prior fractal investigations of the Fitts task use monofractal analysis and diverge across either a “nomothetic” (Wijnants et al., 2012) or “mechanistic” (Slifkin and Eder, 2014) interpretation of monofractal results, the present work uses multifractal analysis and comparison of multifractal results to linear surrogates of the original movement series so as to test directly the nomothetic interpretation of cascade dynamics (e.g., Van Orden et al., 2003; Ihlen and Vereijken, 2010; Stephen and Van Orden, 2012), i.e., specifically to test whether the movement series entailed that Fitts performance was the product of non-linear interactions across time scales.

The present research found that aiming variability in the Fitts task, i.e., the standard deviation of positions of the hand during target contact, was predictable from non-linear interactions across scales at the hand and the head. Crucially, this appeal directly to non-linear interactions across scales follows directly from the facts that aiming variability was not sooner – or to any degree – predictable from the multifractal-spectrum width alone and that the better predictors of aiming variability were t-statistics expressing the difference of the original series from linear surrogates. It is important to note that any monofractal results are indistinguishable from the structure expressed in linear surrogates (Ihlen and Vereijken, 2010; Kelty-Stephen et al., 2013; Wijnants, 2014; Kelty-Stephen and Wallot, 2017). Hence, although this work did not seek to estimate the mechanistic contribution, the use of surrogate testing guarantees that these results are due specifically to non-linear cascade dynamics and not reducible to mechanistic explanations.

The present research further found that the effect of non-linear interactions across scale differed according to different part of the participating movement system. Non-linear interactions across time scale associated with wider multifractal spectra at the hand reduced aiming variability in the Fitts task. Non-linear interactions across time scale associated with wider multifractal spectra at the head reduced aiming variability immediately following closing of the eyes, suggesting that effects of visual feedback depend on the head-sway of the participating movement system. Hence, contrary to Slifkin and Eder (2014), fractality is not merely a symptomatic response to changes in use visual information–on the contrary, multifractality predicts the tensegrity-based usage of visual information. Non-linear interactions across time scale associated with wider multifractal spectra at the head and the hand reduced aiming variability over the entire experimental task for participants who began with the expectation that they might have to close their eyes. Hence, the movement system performs the Fitts task according to an interaction of its expectations of how long visual feedback will be available with its non-linearly cascade-driven head and hand.

In short, aiming in the Fitts task is a full-bodied task that relies on non-linear interactions that span the body no less than the time scales of expectations and explicit instructions. The current work is the latest supporting the notion of motor coordination as a process developing through interactions across multiple scales (e.g., Turing, 1952; Gottlieb, 2007; Molenaar, 2008). This notion appears here in the form of the multifractal tensegrity model (Turvey and Fonseca, 2014), offering early steps toward resolution of Bernstein’s (1967) classic challenges posed by multiple degrees of freedom. The multifractal-tensegrity resolution offered here is that multiple degrees of freedom are what appear to us as the finest details in a system that flow from – but, equally well, collapse under – larger-scaled means (i.e., synergies) for organizing these particulars. Multifractal fluctuations are an essential hallmark of such systems capable of interactions across scales, and estimating the multifractal geometry of movement quantifies the strength of non-linear interactions across scales and thus offers a view into how the movement system engages those degrees of freedom in cross-scale interactions across time. The relevance of the multifractal-tensegrity notion appears here in one of the most fundamental paradigms of movement science, suggesting that the role of multifractal tensegrity extends even to foundational principles of movement that focus so much of the research on goal-directed coordination of the movement system.

## Data Availability

The datasets generated for this study are available on request to the corresponding author.

## Author Contributions

CB contributed to the experimental design, collected all human subjects’ data, and contributed to the data analysis as well as the composition of the manuscript. NC and JZ contributed to the data analysis and the composition of the manuscript. DK-S contributed to the experimental design, data collection, and composition of the manuscript.

## Conflict of Interest Statement

The authors declare that the research was conducted in the absence of any commercial or financial relationships that could be construed as a potential conflict of interest.

## Footnotes

1 https://dx.doi.org/10.17605/OSF.IO/S8BHV
