# Supplementary material for: Non-linear Amplification of Variability Through Interaction Across Scales Supports Greater Accuracy in Manual Aiming: Evidence From a Multifractal Analysis With Comparisons to Linear Surrogates in the Fitts Task
Source: Front Physiol. 2019 Aug 7;10:998. doi: 10.3389/fphys.2019.00998 (PMC6692465; doi:10.3389/fphys.2019.00998)
Supplement: Supplementary file 1 [file Table_1.DOCX]

Supplementary Material

Nonlinear amplification of variability through interaction across scales supports greater accuracy in manual aiming: Evidence from a multifractal analysis with comparisons to linear surrogates in the Fitts task

Christopher A. Bell, Nicole S. Carver, John A. Zbaracki & Damian G. Kelty-Stephen*

*** Correspondence:** Damian G. Kelty-Stephen, foovian@gmail.com

**Supplementary Table 1.** Significant effects from regression modeling of manual-aiming variability SD(Aim).

| Predictor | Coefficient | SE | *p* |
| --- | --- | --- | --- |
| Half | 1.88×10^-3^ | 6.08×10^-4^ | < .01 |
| Mean(Head) | 1.81×10^1^ | 3.60×10^0^ | < .0001 |
| SD(Head) | -5.64×10^0^ | 2.49×10^0^ | < .05 |
| Closedeyes | 7.04×10^-3^ | 2.64×10^-3^ | < .01 |
| Number(Epoch)×Size(Epoch) | 6.13×10^-6^ | 3.39×10^-6^ | .07 |
| Number(Epoch)×Mean(Head) | -2.76×10^-1^ | 1.28×10^-1^ | < .05 |
| Size(Epoch)×Mean(Head) | -1.37×10^-1^ | 5.09×10^-2^ | < .01 |
| Size(Epoch)×SD(Head) | 1.06×10^-1^ | 4.14×10^-2^ | < .05 |
| Closedeyes×Mean(Head) | -1.47×10^1^ | 6.37×10^0^ | < .05 |
| Closedeyes×SD(Head) | 1.14×10^1^ | 6.08×10^0^ | .06 |
| Closedeyes×Number(Epoch) | -1.95×10^-4^ | 9.78×10^-5^ | < .05 |
| Closedeyes×Number(Epoch)×Mean(Head) | 4.39×10^-1^ | 2.29×10^-1^ | .06 |
| Expectation×Mean(Head) | -6.91×10^0^ | 2.41×10^0^ | < .01 |
| Expectation×SD(Head) | 6.43×10^0^ | 1.89×10^0^ | < .001 |
| Expectation×Closedeyes | 9.26×10^-3^ | 1.90×10^-3^ | < .0001 |
| Expectation×Closedeyes×Mean(Head) | -4.11×10^1^ | 5.53×10^0^ | < .0001 |
| Expectation×Closedeyes×SD(Head) | 9.35×10^0^ | 4.35×10^0^ | < .05 |
